# Advocating an attack against severe malaria: a cost-effectiveness analysis

**DOI:** 10.1186/s12889-019-8141-y

**Published:** 2020-01-07

**Authors:** Scott Greenhalgh, Veda Chandwani

**Affiliations:** 1 Department of Mathematics, Siena College, 515 Loudon Road, Loudonville, NY 12211 USA; 2 Department of Biology, Siena College, 515 Loudon Road, Loudonville, NY 12211 USA

**Keywords:** *Plasmodium falciparum*, Malaria, Severe malaria, Cerebral malaria, Anemia, Gut microbiota, Disability adjusted life-years, Incremental cost-effectiveness ratio

## Abstract

**Background:**

A recent study found that the gut microbiota, *Lactobacillus* and *Bifidobacterium,* have the ability to modulate the severity of malaria. The modulation of the severity of malaria is not however, the typical focal point of most widespread interventions. Thus, an essential element of information required before serious consideration of any intervention that targets reducing severe malaria incidence is a prediction of the health benefits and costs required to be cost-effective.

**Methods:**

Here**,** we developed a mathematical model of malaria transmission to evaluate an intervention that targets reducing severe malaria incidence. We consider intervention scenarios of a 2-, 7-, and 14-fold reduction in severe malaria incidence, based on the potential reduction in severe malaria incidence caused by gut microbiota, under entomological inoculation rates occurring in 41 countries in sub-Saharan Africa. For each intervention scenario, disability-adjusted life years averted and incremental cost-effectiveness ratios were estimated using country specific data, including the reported proportions of severe malaria incidence in healthcare settings.

**Results:**

Our results show that an intervention that targets reducing severe malaria incidence with annual costs between $23.65 to $30.26 USD per person and causes a 14-fold reduction in severe malaria incidence would be cost-effective in 15–19 countries and very cost-effective in 9–14 countries respectively. Furthermore, if model predictions are based on the distribution of gut microbiota through a freeze-dried yogurt that cost $0.20 per serving, a 2- to 14-fold reduction in severe malaria incidence would be cost-effective in 29 countries and very cost-effective in 25 countries.

**Conclusion:**

Our findings indicate interventions that target severe malaria can be cost-effective, in conjunction with standard interventions, for reducing the health burden and costs attributed to malaria. While our results illustrate a stronger cost-effectiveness for greater reductions, they consistently show that even a limited reduction in severe malaria provides substantial health benefits, and could be economically viable. Therefore, we suggest that interventions that target severe malaria are worthy of consideration, and merit further empirical and clinical investigation.

## Background

Sub-Saharan Africa suffers the vast majority of the world’s malaria burden, with an estimated 92% of incidences occurring annually [1]. Due to this disproportionate malaria burden, great effort is underway to develop and scale-up malaria interventions, with the ultimate goal to reduce the entire world’s malaria burden to zero. To date, the primary strategy of most malaria interventions focuses on some form of transmission blocking, whether it be with fungal insecticides [2], increased access to high quality antimalarial drugs [3], the distribution of bed nets [4], or minimizing recurrent malaria incidence in at-risk demographics [5–7]. While these malaria interventions all stand to improve public health, a common theme among them is that they do not outright target one of the highest contributors to the burden of malaria, namely, individuals that suffer some form of severe malaria.

Severe malaria is one of two main classifications for malaria disease [8], with the other typically being referred to as uncomplicated malaria. Severe malaria occurs when serious complications arise during infection, such as cerebral malaria, acute respiratory distress syndrome, low blood pressure, acute kidney injury, hypoglycemia, and severe anemia, to name but a few [1]. While there are many factors that correlate with the risk of severe malaria, including parasite virulence level and host inflammation, a leading indicator for a severe malaria incidence is parasite burden [9]. In fact, while a high parasite burden is not synonymous with severe malaria incidence, a low parasite burden yields little to no risk for severe malaria [9].

Recently a study found that parasite burden is dramatically reduced by gut microbiota [10]. Specifically, the gut microbiota, *Lactobacillus* and *Bifidobacterium*, are associated with up to a 14-fold decrease in parasite burden [10], and substantially reduce the likelihood of severe malaria. Given this result, we seek to determine the conditions required for an intervention that targets severe malaria to be cost-effective. To do this, we first quantify the health benefits and costs required for an intervention that targets severe malaria to be cost-effective, and then determine whether the distribution of gut-microbiota, through a freeze-dried yogurt, has the potential to be a cost-effective malaria intervention.

To accomplish these goals we developed a mathematical model of malaria transmission calibrated to the malaria transmission intensities, as characterized by the entomological inoculation rate (EIR), of 41 countries in sub-Saharan Africa. Using this model, we evaluate the health benefits and cost-effectiveness of interventions that reduce severe malaria, as measured by disability-adjusted life years (DALYs) averted [11] and the incremental cost-effectiveness ratio (ICER) [12]. We consider intervention scenarios that reflect the average predicted reduction caused by gut microbiota (a 14-fold reduction) [10], the lower bound on the predicted reduction caused by gut microbiota (7-fold reduction) [10], and illustrate that even a 2-fold reduction in severe malaria still holds merit.

## Methods

To estimate the reduction in severe malaria incidence required for an intervention to be cost effective, or very cost effective, we developed a mathematical model of malaria transmission calibrated to the malaria transmission intensities and population demographics of 41 sub-Saharan Africa countries. As the complete prevention of severe malaria incidence is unlikely, at least until the time that malaria eradication is feasible, we base the modulation of malaria severity on recent data of the effects of gut microbiota on parasite burden in mice [10]. In addition, we only consider the effect of the intervention scenarios on malaria incidence reported to healthcare settings and classified as a severe malaria incidence in accordance to WHO standards [1]. Based on these data, we consider interventions that cause 2-, 7-, and 14-fold reductions in severe malaria incidence over the course of a 5-year time horizon. We consider the absence of any reduction in severe malaria incidence as the baseline scenario for our analysis. From these intervention scenarios, the outcomes measured include annual severe malaria incidence averted, the years of life lost due to malaria and years lived with disability because of malaria, as measured through DALYs averted [11], and the cost-effectiveness of the intervention, as measure by ICER [12]. To classify an intervention as cost-effective or very cost-effective, we apply the WHO-CHOICE criterion for cost-effective and very cost-effective interventions in relation to the GDP per capita for each country [13].

### The mathematical model

The developed mathematical model of malaria transmission considers an approach [14] that divides the population into six parts: susceptible individuals (*S*), infected individuals with clinical disease (*D*), asymptomatically infected individuals (*A*), individuals with present, but not detectable, subpatent infection (*U*), treated individuals (*T*), individuals using prophylaxis (*P*) [14–16], susceptible mosquitoes (*M*_*s*_), and infected mosquitoes (*M*_*i*_). The rate susceptible individuals acquire malaria, *λ*, and the rate susceptible mosquitoes acquire infection, *λ*_*M*_, are given by the forces of infection [17, 18]:

1 $$ \lambda = c\alpha \frac{M_i}{N},\kern0.5em \mathrm{and}\kern1.5em {\lambda}_M= c\beta \frac{I}{N}. $$

Here *c* is the mosquito biting rate, *α* is the mosquito-to-human transmission probability, *β* is the human-to-mosquito transmission probability, 1 ∕ *d*_0_ is the mean mosquito lifespan, *I* is the total number of humans infected with malaria, and *N* is the human population size. The mathematical model also considers the probabilities of symptomatic infection, *ϕ*, and effective treatment of clinical malaria, *f*_*T*_, in addition to the rates of recovery from clinical malaria, *r*_*T*_, asymptomatic malaria, *r*_*A*_, severe malaria, *r*_*D*_, the period of protection provided by prophylaxis, 1 ∕ *r*_*P*_, and the clearance rate of sub-patent infection, *r*_*U*_ [14–16].

We parameterize our mathematical model according to one of the main measures of malaria transmission intensities, the EIR. The EIR is the number of infectious bites of malaria per person per year (ibpppy). We consider EIR values for 41 countries in sub-Saharan African (Additional file 1: Table S1), which range from 0.05 to 220 ibpppy [19]. These EIR estimates, with the assumption that the mosquito population is at equilibrium, and the transmission probabilities between humans and mosquitoes (Table 1) allows us to estimate the mosquito to human ratio for each considered country. In addition, to estimate the proportion of malaria incidence that is severe, we use published data on malaria incidence and severe malaria incidence reported to health care providers [31] (Additional file 1: Table S1). We use these country specific estimates of the proportion of malaria incidence that is severe (Additional file 1: Table S1), together with the predicted trajectory of malaria incidence under each countries’ EIR to evaluate the considered intervention scenarios. Further details of the model parameters and model equations are available in Table 1 and Additional file 1.

**Table 1:** Parameters values, distributions, and sources

Symbol	Parameter	Base value	Distribution	Citation
*c*	Mosquito biting rate	1/3 day^−1^	Exp(3)	[20]
*α*	Transmission probability (mosquito to human)	0.25	*N*(0.25,0.04)	[16]
*β*	Transmission probability (human to mosquito)	0.433	*Beta*(12.5,16.35)	[21]
*m*	Mosquito to human ratio	1 − 3.8		Fit
*r*_*T*_	Recovery rate for clinical malaria (with chemotherapy)	1/21 day^−1^		[16]
*r*_*D*_	Recovery rate for severe malaria (without chemotherapy)	1 ∕ 180 day^−1^		[16]
*r*_*A*_	Recovery rate from asymptomatic malaria	1 ∕ 180 day^−1^		[16]
1 *∕ r*_*P*_	Duration of post-treatment prophylaxis effect	28 days	U[21, 35]	[16]
*r*_*U*_	Clearance rate of sub-patent infection	1 ∕ 180 day^−1^		[16]
1 *∕ d*_0_	Mean mosquito life span	7.6 days	log*N*(1.98,0.31)	[14]
*ϕ*	probability of symptomatic incidence	0.5		[16]
*f*_*t*_	probability clinical malaria is effectively treated	0.5	0.05 *to* 1	[14, 16]
*ω*	Proportion reporting for treatment to a healthcare setting	0.618	Beta(65.4,40.42)	[22]
*ψ*	Reduction in severe malaria incidence	–	0, 1/2, 1/7, 1/14	[10]
*r*	DALY discount rate	0.03		[23]
*D*_*M*_	Disability weight of a malaria incidence	0.2078		[23]
*D*_*S*_	Disability weight of a severe malaria incidence	0.133		[24]
*D*_*N*_	Disability weight of neurological sequelae	0.471		[23]
*D*_*A*_	Disability weight of severe malaria anemia	0.149		[24]
*D*_*C*_	Disability weight of cerebral malaria	0.471		[25]
*D*_*CA*_	Disability weight of cerebral malaria and severe malarial anemia	0.620		[25]
*D*_*NA*_	Disability weight of neurological sequelae and severe malarial anemia	0.483		[23]
*D*_*D*_	Disability weight of death	1.0		[23]
*L*_*M*_	Duration of malaria incidence	5.1 days		[26]
*L*_*S*_	Duration of severe malaria incidence	8.75 days		[27]
*L*_*N*_	Duration of neurological sequelae	10.1 days		[28]
*L*_*A*_	Duration of severe malarial anemia	11 days		[26]
*L*_*C*_	Duration of cerebral malaria	6.5 days		[27]
*L*_*CA*_	Duration of cerebral malaria and severe malarial anemia	11 days		[5]
*L*_*NA*_	Duration of neurological sequelae and severe malarial anemia	11 days		[5]
*L*_*D*_	Years of life lost in death	55 years		[29]
*R*_*M*_	Risk of malaria infection	0.9943		[30]
*R*_*S*_	Risk of severe malaria given malaria infection	0.0057		[30]
*R*_*N*_	Risk of neurological sequelae given severe malaria	0.098		[28]
*R*_*A*_	Risk of severe malarial anemia given severe malaria	0.322	U[0.043,1]	[31]
*R*_*C*_	Risk of cerebral malaria given severe malaria	0.002		[30]
*R*_*CA*_	Risk of cerebral malaria and severe malarial anemia given severe malaria infection	0.00096		[30]
*R*_*NA*_	Risk of neurological sequelae and severe malarial anemia given severe malaria infection	0.0316		[28, 31]
*death*_*S*_	Risk of death due to severe malaria			Additional file 1: Table S1
*death*_*N*_	Risk of death due to neurological sequelae	0.1835		[32]
*death*_*A*_	Risk of death due to severe malarial anemia	0.097		[32]
*death*_*C*_	Risk of death due to cerebral malaria	0.192		[30]
*death*_*CA*_	Risk of death due to cerebral malaria and severe malarial anemia	0.1835		[33]
*death*_*NA*_	Risk of death due to neurological sequelae and severe malarial anemia	0.347		[23]
*υ*	Cost of a uncomplicated malaria incidence	5.84	Tri(2.36, 3.50, 23.65)	[34]
*σ*	Cost of a severe malaria incidence	30.26	Tri(15.64, 19.14, 137.87)	[34]
*δ*	Cost per serving of yogurt		Tri(0.2,0.245,0.29)	[35]
*θ*	Distribution costs per serving	0.075	U(0.06,0.09)	[36]
*Y*	Servings of yogurt consumed per week	2.27	U(2.21,2.33)	[37]

### The intervention

We considered an intervention that targets reducing severe malaria incidence over a 5-year period in order to illustrate the merit of such interventions for clinical studies. The intervention is based upon modulating the severity of malaria, as recent studies illustrate the potential to accomplish such a feat through the promotion of a microbiome that includes the microbiota, *Lactobacillus* and *Bifidobacterium* [10]. Specifically, the microbiota, *Lactobacillus* and *Bifidobacterium,* are associated with a 14-fold reduction in parasite burden [38]*.* So, we evaluate up to a 14-fold reduction in severe malaria incidence to determine the per person costs so that such an intervention is cost-effective or very cost-effective.

To conduct such an evaluation, we parameterized our model with freely available demographic data of the considered 41 countries in sub-Saharan Africa [39], and published data on the malaria transmission intensity, as described by the EIR [19], for each respective country.

### Intervention costs

The treatment of uncomplicated malaria is assumed to correspond to the WHO recommended guidelines for first-line treatment of uncomplicated *Plasmodium falciparum* malaria [40]. The treatment of uncomplicated malaria typically corresponds to the use of an artemisinin-based combination therapy, such as artemether-lumefantrine, over the course of a 3 day treatment period [40], with a median cost of $5.84 USD [34]. Similarly, we also assume that the treatment of severe malaria corresponds to WHO recommended guidelines [40] with estimated median costs to treat an incidence of severe malaria of $30.26 USD [34].

For an intervention based on the ability of gut microbiota to modulate malaria severity [10], we also consider the costs associated to the distribution of gut microbiota through a freeze-dried yogurt [41]. Specifically, we consider intervention costs based on yogurt prices of $0.20–0.29 USD for a 4–6 oz serving [35], along with estimates that 2.27 servings of yogurt are consumed per week [37]. In addition, we assume that the distribution costs associated to the distribution of the freeze-dried yogurt are in line with the $0.06–0.09 per unit cost for the distribution of antimalarial drugs [36].

### Intervention effectiveness

We quantified the effectiveness of the intervention that targets reducing severe malaria incidence in terms of Disability Adjusted Life Years (DALYs), which is a common measure of the health burden resulting from years of life lost and years lived with disability [42–44]. We calculated time-discounted DALYs lost to malaria, severe malaria, cerebral malaria, neurological sequelae, and severe malaria anemia (Table 1). Annual DALYs averted were calculated by subtracting each intervention scenario from the base scenario for each respective country.

### Intervention cost-effectiveness

We calculated the per person costs so that the proposed intervention would qualify as cost-effective or very cost-effective under the malaria transmission settings occurring in the 41 considered countries in sub-Saharan Africa. For each of these countries, we obtained GDP per capital estimates [45] to determine the willingness-to-pay for a i) cost-effective intervention, and ii) a very cost-effective intervention. To do so, we made use of the incremental cost-effectiveness ratio (ICER),

2 $$ ICER=\frac{\Delta C}{\Delta D} $$

where Δ*D* is the annual DALYs averted per person, relative to the baseline intervention, and Δ*C* = *C*_1_ − *C*_0_ is the change in the average cost of a malaria incidence per person. Here, *C*_1_ is the average cost of a malaria incidence per person under the intervention, and *C*_0_ is the average cost of a malaria incidence per person under the base line scenarios, respectively. Furthermore, the average costs of a malaria incidence per person are determine by the reduction factor *ψ*, the average cost of an uncomplicated malaria incidence *υ*, and the average cost of a severe malaria incidence *σ*:

3 $$ {\mathrm{C}}_0=\upsilon x+\sigma \left(1-x\right), and\ {\mathrm{C}}_1=\upsilon x+\sigma \left(1-\psi \right)\left(1-x\right)+\upsilon \psi \left(1-x\right)+g $$

where *x* is the proportion of incidence that are uncomplicated and *g* is the per person cost of the gut microbiota intervention.

In accordance with the WHO standards [46], a cost-effective intervention for a country occurs when ICER ≤ 3GDP, and a very cost-effective intervention occurs when ICER ≤ GDP. Thus, from (3) it follows that the per person cost of a cost-effective intervention involving gut microbiota must satisfy

4 $$ g<3\cdotp GDP\ \Delta D+\psi \left(\sigma -\upsilon \right)\left(1-x\right), $$

and the per person cost of a very cost-effective intervention must satisfy

5 $$ g< GDP\ \Delta D+\psi \left(\sigma -\upsilon \right)\left(1-x\right). $$

### Sensitivity analysis

To quantify the contribution of parameters to the variability of predicted outcomes, we calculated first-order sensitivity indices [47]. First-order sensitivity indices indicate how uncertainty in each parameter contributes to the variability of model outcomes. Details of the parameters and probability distributions used in this calculation are available in Table 1.

## Results

We evaluated the health benefits of an intervention that reduces severe malaria incidence, and identified the thresholds for such an intervention to be cost-effective or very cost-effective for 41 countries in sub-Saharan Africa. Furthermore, we evaluate an intervention that targets severe malaria based on the costs and effects associated to the distribution of gut microbiota through a freeze-dried yogurt, finding that such an intervention is likely cost-effective in at least 25 countries in sub-Saharan Africa.

**Fig. 1:**
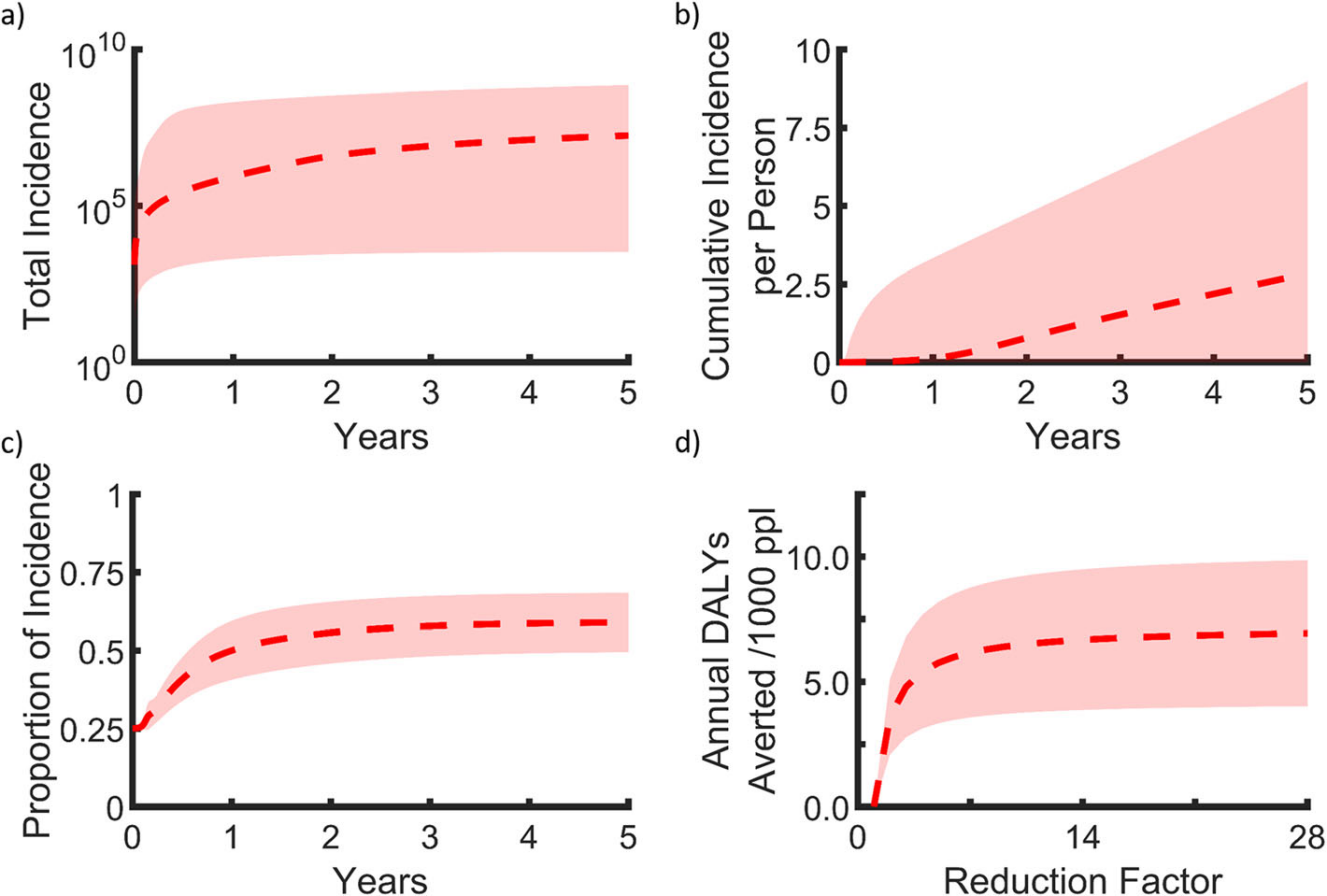
Malaria incidence and DALYs over a period of five years. **a** Predicted malaria incidence for the 41 considered countries in sub-Saharan Africa, **b** total malaria incidence for the 41 considered countries in sub-Saharan Africa, **c** the proportion of malaria incidence reported to a healthcare provider, and **d** annual DALYs averted for given reduction factor. The mean for all countries (red line), and 95% quantiles (shaded region)

Our model predicted a total of 1.8 × 10^7^ (3.3 × 10^3^ − 7.3 × 10^8^ ) malaria incidence over the course of a 5-year period (Fig. 1a), which translates to 3.2 (0.001 − 8.4) total incidence per person per year (Fig. 1b). These predictions are within current estimates of the malaria incidence for each respective country, given recent trends on malaria transmission intensities [31]. Furthermore, the predicted proportion of severe malaria incidence was also in line with the literature [5, 6, 22], as simulations place this proportion at 0.59 (0.49 − 0.68) (Fig. 1c). Given these baseline values of malaria incidence, our model predicted that 0.24–44.0, 0.01–40.91, and 0.00–23.87 annual incidences of severe malaria per 1000 people would be averted for 14-fold, 7-fold, and 2-fold reduction factors, respectively, depending on malaria transmission intensity and population demographics (Table 2, Fig. 1d).

**Table 2:** Severe malaria incidence, deaths, and DALYs averted

Scenario	Annual severe malaria incidence averted per 1000 ppl	Annual malaria deaths averted per 1000 ppl	Annual DALYs saved per 1000 ppl
2-fold reduction	9.2 (0.00–23.87)	0.7 (0.00–3.28)	3.6 (0.00–20.35)
7-fold reduction	15.7 (0.01–40.91)	1.1 (0.00–5.63)	6.2 (0.00–34.88)
14-fold reduction	17.0 (0.24–44.00)	1.2 (0.00–6.09)	6.7 (0.00–37.79)

Entries correspond to averages with the range of values for all 41 countries in parentheses

**Fig. 2:**
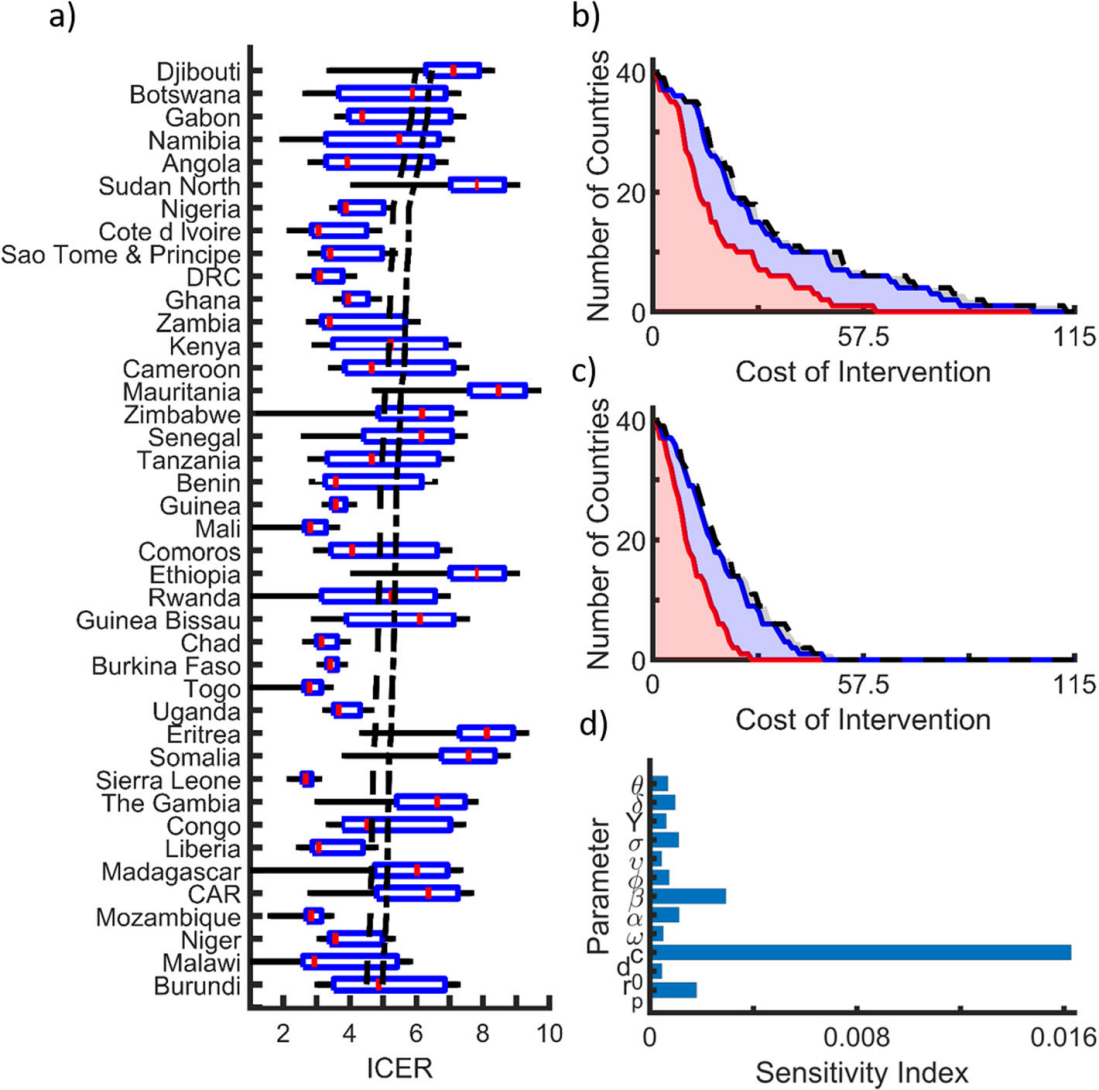
ICER, intervention cost and effectiveness, and sensitivity of cost-effectiveness to model parameters. **a** Boxplots of ICER values based on sample sizes of 10,000 stochastic parameter samples with threshold lines for a cost-effective intervention (black dash dot line) and a very cost-effective intervention (black dashed line), respectively. **b** Per person intervention costs for a cost-effective intervention and **c** Per person intervention costs for a very cost-effective intervention. Colored regions correspond to a 14-fold reduction in severe malaria incidence (black), 7-fold reduction in severe malaria incidence (blue), and 2-fold reduction in severe malaria incidence (red). **d** First order sensitivity indices for average ICER. Calculations are based on sample sizes of 10,000, where the reduction factor of severe malaria incidence is *ϕ*~*U*[2, 14]

For the costs and effects associated to the distribution of gut microbiota through a freeze-dried yogurt, we found the median ICER ranged from 4.7 to 3.5 × 10^6^ across all countries. When considering specific countries, our findings show that a gut microbiota intervention would be cost-effective in 29 of 41 sub-Saharan African countries (Fig. 2a). Furthermore, of these 29 countries, 16 have their entire interquartile range of predicted ICER values below the cost-effectiveness threshold of 3 times the GDP per capita (Fig. 2a). Concerning the potential for a very cost-effective intervention, 25 countries fall below the threshold of the GDP per capital, with 14 of the 25 countries having their interquartile range for predictions of the ICER below the very cost-effective threshold of the GDP per capita. In addition, such reductions in severe malaria incidence would also avert between 0.001–6.09 deaths per 1000 people and 0.001–37.19 annual DALYs per 1000 people (Table 2), depending on the intervention reduction factor and the EIR. Given these results, the upper cost threshold for such an intervention to be cost-effective in at least one country is $112 USD per person annually, and $49 USD per person annually to be very cost-effective (Fig. 2b-c). These numbers improve to 15–19 countries for cost-effective interventions and 9–14 countries for very cost-effective interventions (Fig. 2b-c) when costs are assumed to be in line with the upper cost for an uncomplicated malaria incidence of $23.65 USD (Table 1) to the average cost of severe malaria incidence of $30.26 USD (Table 1).

With regard to model predictions, our sensitivity analysis showed that the largest contributor to the variation in the ICER was uncertainty in the mosquito biting rate, followed by the human to mosquito transmission probability (Fig. 2d). In addition, the sensitivity analysis also showed that the effect of uncertainty in intervention costs were in the same order of magnitude as most other model parameters (Fig. 2d).

## Discussion

To date, the vast majority of malaria interventions do not outright target severe malaria. While the reduction of severe malaria incidence may not directly cause malaria eradication, severe malaria is responsible for substantial health and economic burdens. Our results indicate that reducing severe malaria through interventions, such as the distribution of the gut microbiota, *Lactobacillus* and *Bifidobacterium,* by means of a freeze-dried yogurt, may be a cost-effective strategy for areas seeking malaria control.

Our predictions illustrate that an intervention that causes a 2- to 14-fold reduction in severe malaria incidence is potentially cost-effective in the majority of the considered sub-Saharan African countries. Furthermore, these predictions are likely conservative, as reducing severe malaria incidence would decrease the average duration of malaria infection, and subsequently decrease transmission intensity. Likewise, our model predictions for a gut microbiota intervention are also likely conservative. To elaborate, the distribution of gut microbiota through a freeze-dried yogurt would also promote and restore healthy gut microbiomes, which may help fend off diarrheal disease, decrease cholesterol [48], and convey multiple other long-term health benefits [49]. Furthermore, freeze-dried yogurt can carry nutritional value, and thereby may aid in reducing a large at-risk group for malaria infection, namely, malnourished children [50, 51].

The predictions of annual malaria incidence by our model are consistent with previously published data [14, 52, 53]. In addition, the predicted health benefits are comparable with current estimates of other interventions, such as the DALYS averted by the use of HIV protease inhibitors to prevent recurrent malaria incidence in HIV infected children [5, 6] and prevention campaigns [54]. With respect to intervention costs, although higher costs per person unsurprisingly reduced the number of countries where a severe malaria intervention is cost-effective, our estimates of the ICER are comparable to various scale-up programs [55], and the effects of media on promoting life-saving practices [56].

Our study faces several potential limitations. To begin, our model only considered stable transmission intensities of 0.05 to 220 ibpppy, and did not include seasonal increases that often occur in many sub-Saharan African countries. In addition, our model does not account for age structure or the various kinds of malaria immunity. Furthermore, the maximum reduction in severe malaria incidence (i.e., the 14-fold and 7-fold reductions) is based on gut microbiota studies of mice, although we also provide estimates that even a 2-fold decrease in severe malaria provides substantial benefits. Finally, our decision criteria of cost-effective and very cost-effective interventions is based on the WHO-CHOICE recommendations with respect to country specific GDP [57], and therefore does not provide information on the affordability or feasibility of such interventions [58].

In this study, we estimate the reduction in the health burden of malaria caused by reducing severe malaria incidence for stable malaria transmission in 41 sub-Saharan African countries. As such, our model is easily adaptable to describe other malaria transmission settings. For instance, modifications to incorporate seasonal transmission, and a more selective distribution schedule for the gut microbiota, would only require the inclusion of periodic parameters. The likely result of such modifications would be an even more cost-effective intervention. In the same vein, modifications to include an age structure would also likely provide positive results, as the distribution of gut microbiota through freeze-dried yogurt would be more effective through targeting children, as they typically endure more severe and more frequent malaria infections.

## Conclusion

In summary, to inform the potential design of malaria interventions that target severe malaria, we developed a mathematical model to predict the health benefit and cost-effectiveness of 2-, 7-, and 14-fold reductions in severe malaria incidence. Our analyses indicates that the health and economic savings of even a 2-fold reduction in severe malaria incidence would be substantial. Consequently, we suggest that interventions that target severe malaria could be economically viable and beneficial to health, and thus further empirical research on the possibility of such interventions merits consideration.

## Supplementary information

**Additional file 1.** Supplementary materials for advocating an attack against severe malaria. The supplementary materials contains details of the malaria transmission model, proportion of malaria incidence reported as severe in healthcare settings, and demographic data for the 41 considered sub Saharan African countries.

## Data Availability

All data generated or analyzed during this study are included in this published article [and its supplementary information files.

## Abbreviations

DALYs: Disability adjusted life-years
EIR: Entomological inoculation rate
GDP: Gross domestic product
ibpppy: Infectious bites per person per year
ICER: Incremental cost-effectiveness ratio
USD: United States Dollars
WHO: World Health Organization
