# Supplementary material for: Advocating an attack against severe malaria: a cost-effectiveness analysis
Source: BMC Public Health. 2020 Jan 7;20:17. doi: 10.1186/s12889-019-8141-y (PMC6947859; doi:10.1186/s12889-019-8141-y)
Supplement: Supplementary file 1 — Additional file 1. Supplementary materials for advocating an attack against severe malaria. The supplementary materials contains details of the malaria transmission model, proportion of malaria incidence reported as severe in healthcare settings, and demographic data for the 41 considered sub Saharan African countries. [file 12889_2019_8141_MOESM1_ESM.pdf]

Supplementary material for advocating an attack against severe malaria

Scott Greenhalgh, Veda Chandwani

This appendix provides further details on the mathematical model of malaria transmission, in addition to data used in the cost effectiveness analysis.

Model Equations

The population is divided into six distinct parts: susceptible individuals (S), individuals with the clinical disease (D), asymptomatic individuals (A), individuals with the infection sub-patent – present but not detectable – (U), treated individuals (T), individuals using prophylaxis (P), susceptible mosquitoes (M_s), and infected mosquitoes (M_i). The transition between the model compartments is governed by:

$$\frac{dS}{dt} = -\lambda S + r_P P + r_U U,$$

$$\frac{dD}{dt} = \lambda\phi(1 - f_T)(S + A + U) - r_D D,$$

$$\frac{dA}{dt} = \lambda(1 - \phi)(S + U) - \lambda\phi A + r_D D - r_A A,$$

$$\frac{dU}{dt} = -\lambda U + r_A A - r_U U,$$

$$\frac{dT}{dt} = \lambda\phi f_T(S + A + U) - r_T T,$$

$$\frac{dP}{dt} = r_T T - r_P P,$$

$$\frac{dM_s}{dt} = d_0 M - \lambda_M M_s - d_0 M_s,$$

$$\frac{dM_i}{dt} = \lambda_M M_s - d_0 M_i,$$

where

$$\lambda = c\alpha\frac{M_i}{N}, \qquad \text{and} \qquad \lambda_M = c\beta\frac{I}{N}.$$

References

1. Penny MA, et al. (2015) Distribution of malaria exposure in endemic countries in Africa considering country levels of effective treatment. *Malar J* 14(1):384.
2. Camponovo F, Bever CA, Galactionova K, Smith T, Penny MA (2017) Incidence and admission rates for severe malaria and their impact on mortality in Africa. *Malar J* 16(1):1.
3. World Bank (2018) *GDP per capita, PPP (current international $)*.

Table S1. EIR, proportion of malaria incidence that are severe, GDP, and population.

Country	EIR[a]	Proportion severe[b]	GDP[c]	Population[c]
Angola	49.8	0.07	6454.1	28813460
Benin	72.8	0.54	2167.6	10872300
Botswana	5.3	0.60	16956.7	2250600
Burkina Faso	118.1	0.29	1771	16646430
Burundi	13.5	0.02	777.8	10542120
Cameroon	67.4	0.45	3609.4	23439190
Central African Republic	61.4	0.32	698.7	4594620
Chad	27.8	0.07	1990.7	14452540
Comoros**	46.5	0.34	1521.9	75600
Congo	49	0.28	5717.3	5125820
Cote d’ivoire	78.8	0.50	3693.4	23695920
Democratic Republic of Congo	47.4	0.24	801.6	78736150
Equatorial Guinea	76.8	0.31	26058.1	1221490
Ethiopia	1	0.29	1734.5	102403200
Gabon	71.7	0.28	18102.9	1979790
Ghana	52.3	0.42	4292.4	28206730
Guinea	39.7	0.18	1966.4	12395920
Guinea Bissau	6.3	0.13	1608.7	1815700
Kenya	7.7	0.68	3155.1	48461570
Liberia	60.2	0.29	812.7	6293250
Madagascar	42	0.33	1506.2	24894550
Malawi	54.5	0.13	1168.8	18091580
Mali	76	0.30	2125.7	17994840
Mauritania	5.4	0.07	3852.5	4301020
Mozambique	65.8	0.64	1216.8	28829480
Namibia	11.3	0.48	10624.9	2479710
Niger	35.3	0.29	986.2	20672990
Nigeria	65.7	0.29	5861.1	185989640
North Sudan	7	0.26	4730.5	39578830
Rwanda	2.2	0.09	1912.9	11917510

Sao Tome Principe**	25.8	0.45	3237.4	199910
Senegal	5.8	0.12	2566.1	15411610
Sierra Leone	61	0.07	1476.2	7396190
Tanzania	25.1	0.26	2786.3	55572200
The Gambia	7.3	0.48	1676.9	2038500
Togo	58.9	0.13	1490.5	7606370
Uganda	89.7	0.40	1819.4	41487960
Zambia	26.9	0.13	3933.1	16591390
Zimbabwe	2.8	0.16	2027.1	16150360

**-proportion serve estimate is based country specific EIR and interpolation between EIR and proportion severe data

[a] –values obtained from (1),
[b] –values obtained from (2),
[c] –values obtained fron (3)
